# Chronic Debilitating Headache in Adults Caused by Craniocerebral Disproportion: Treatment by Cranial Vault Expansion

**DOI:** 10.7759/cureus.2187

**Published:** 2018-02-13

**Authors:** Ken R Winston, Brooke French, Jason Bunn

**Affiliations:** 1 Department of Neurosurgery, University of Colorado School of Medicine; 2 Department of Surgery (plastic Surgery), University of Colorado School of Medicine; 3 School of Medicine, University of Colorado School of Medicine

**Keywords:** cranial vault expansion, craniocerebral disproportion, craniostenosis, distraction osteogenesis, hydrocephalus, shunt failure, slit-ventricle syndrome

## Abstract

Craniocerebral disproportion is rarely considered as a cause for chronic, debilitating headache in adults. Children reported with this disorder typically suffer from headaches and lethargy for many years and have multisutural synostosis. The terms craniocerebral disproportion, craniostenosis, and slit-ventricle syndrome are used inconsistently as diagnostic designations. Three adults with craniocerebral disproportion who had been treated in infancy for two different pathologies are reported. All benefited greatly from cranial vault expansion.

## Introduction

A cranial vault that is too small to safely and satisfactorily accommodate a patient’s normal intracranial contents, regardless of circumference and cause, is designated craniocerebral disproportion. It may be caused by multisutural synostosis, either syndromic or occurring following surgery for craniosynostosis or secondary to long-term exposure to low intracranial pressure in infancy [1-8]. Although initially inconsequential, the normal growth of brain continues after cessation of the cranial vault’s ability to expand and thereby results in progressive compromise of cerebrospinal fluid (CSF)-containing spaces and then tight compaction of brain. Craniocerebral disproportion either rarely occurs or is perhaps only rarely recognized in adults [6]. This report presents adults with craniocerebral disproportion who benefited from cranial vault expansion.

## Materials and methods

Clinical material

All adults with hydrocephalus who underwent cranial vault expansion in the University of Colorado Hospital to address craniocerebral disproportion are the basis for this report. This investigation was approved by the Colorado Multi-institutional Review Board (16-1050).

Methods

The details of the technique used for cranial vault expansion in adults were identical to those previously described for children by the senior author [9]. The technique used was expansion by distraction osteogenesis.

## Results

Case 1

**Figure 1:**
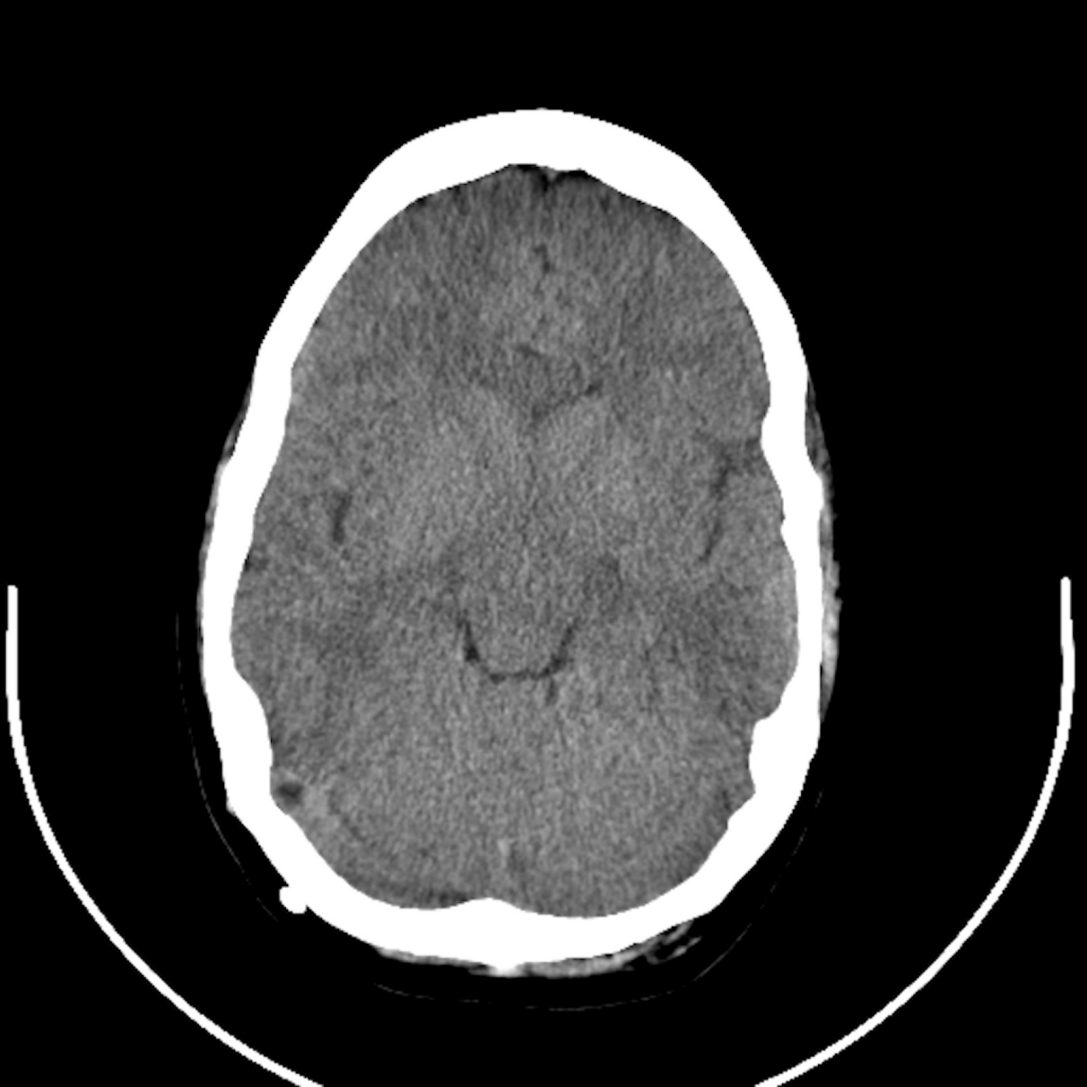
Case 1 pre-expansion

A newborn girl with congenital hydrocephalus due to intracranial hemorrhage during the early postpartum period had a ventriculoperitoneal shunt implanted early in the first year of life. During her teens and early adulthood, she frequently suffered headaches that progressed in both severity and frequency. Neuroimaging repeatedly demonstrated small ventricles (Figure 1). Most of the approximate 40 revisions of her CSF shunt system were attributed to ventricular catheter occlusions. Valves with low outflow resistance were consistently used. Some of these surgeries offered temporary relief of symptoms but many resulted in little or no recognized benefit.

**Figure 2:**
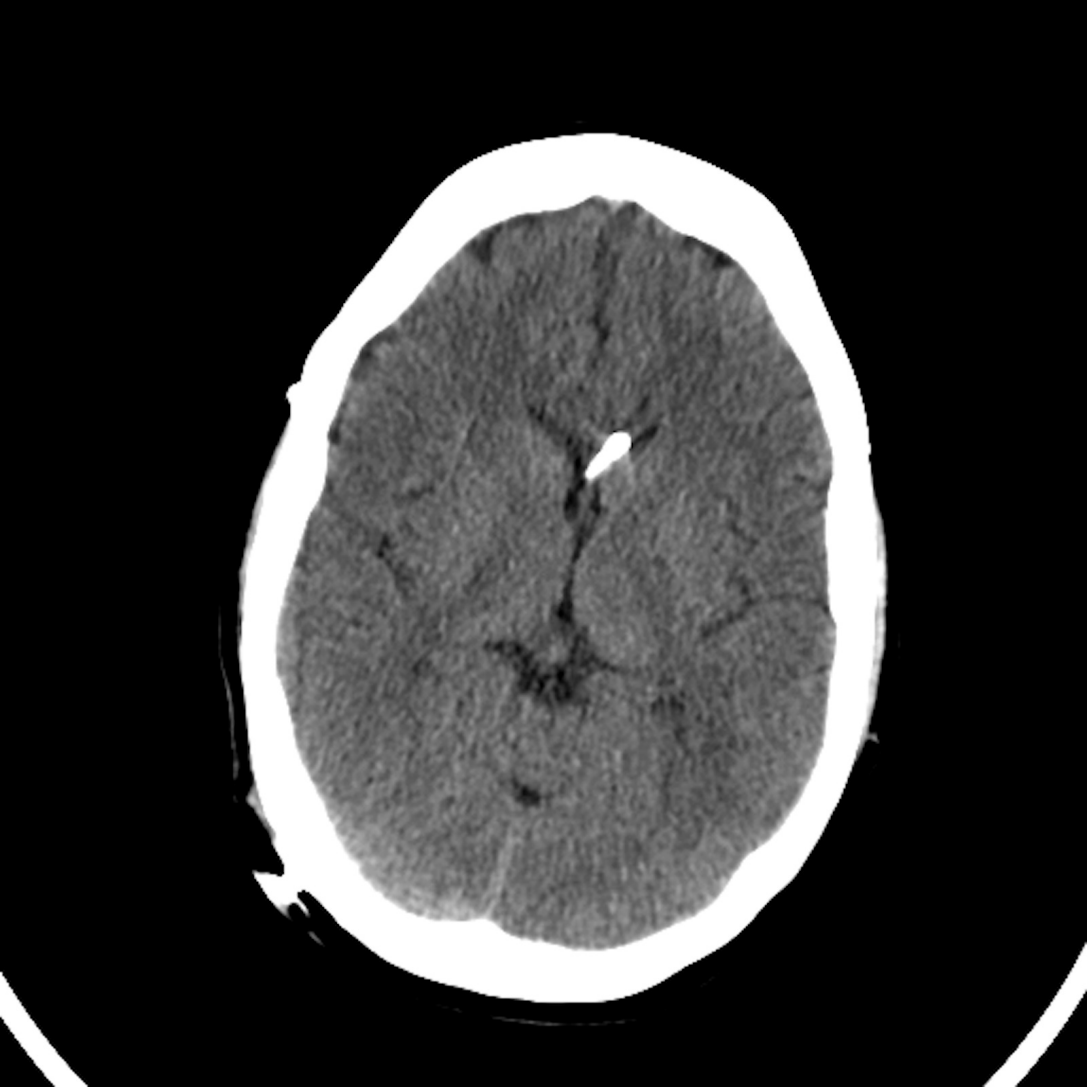
Case 1 post-expansion

Hypothyroidism, factor V Leiden deficiency, intestinal atresia, and a patent atrioventricular (AV) ductus were subsequently diagnosed. At 30 years of age the patient underwent cranial vault expansion by distraction osteogenesis. Both frontoparietal regions were expanded by 1.5 centimeters (cm) over a distraction period of 15 days and a consolidation period of 39 days (Figure 2). Infection at the site of rod penetration was treated with local antibiotics and, at the time of distractor removal, with local debridement of the pin sites.

All pre-expansion headaches and lethargy cleared during the distraction process and have not recurred over the following eight years. She has required three shunt revisions over that time but none in the past four years. The patient and family describe mental and physical functions as being normal.

Case 2

A male child born at 27 weeks gestation developed hydrocephalus secondary to grade 4 intraventricular hemorrhage and a ventriculoperitoneal shunt was implanted at three months of age. Asthma, epilepsy, and Asperger’s syndrome were diagnosed over the following years. Frequent, worsening and generalized headaches began in his teen years and the patient underwent approximately 30 shunt revisions. Most of these surgeries provided little significant relief from his constant headaches that were independent of body position. After a proximal shunt revision at 23 years of age, the patient had a severe decline in neurologic status manifested by posturing and several days of minimal responsiveness. He regained consciousness over seven to ten days but experienced near-constant, severe generalized headaches for the next three years.

**Figure 3:**
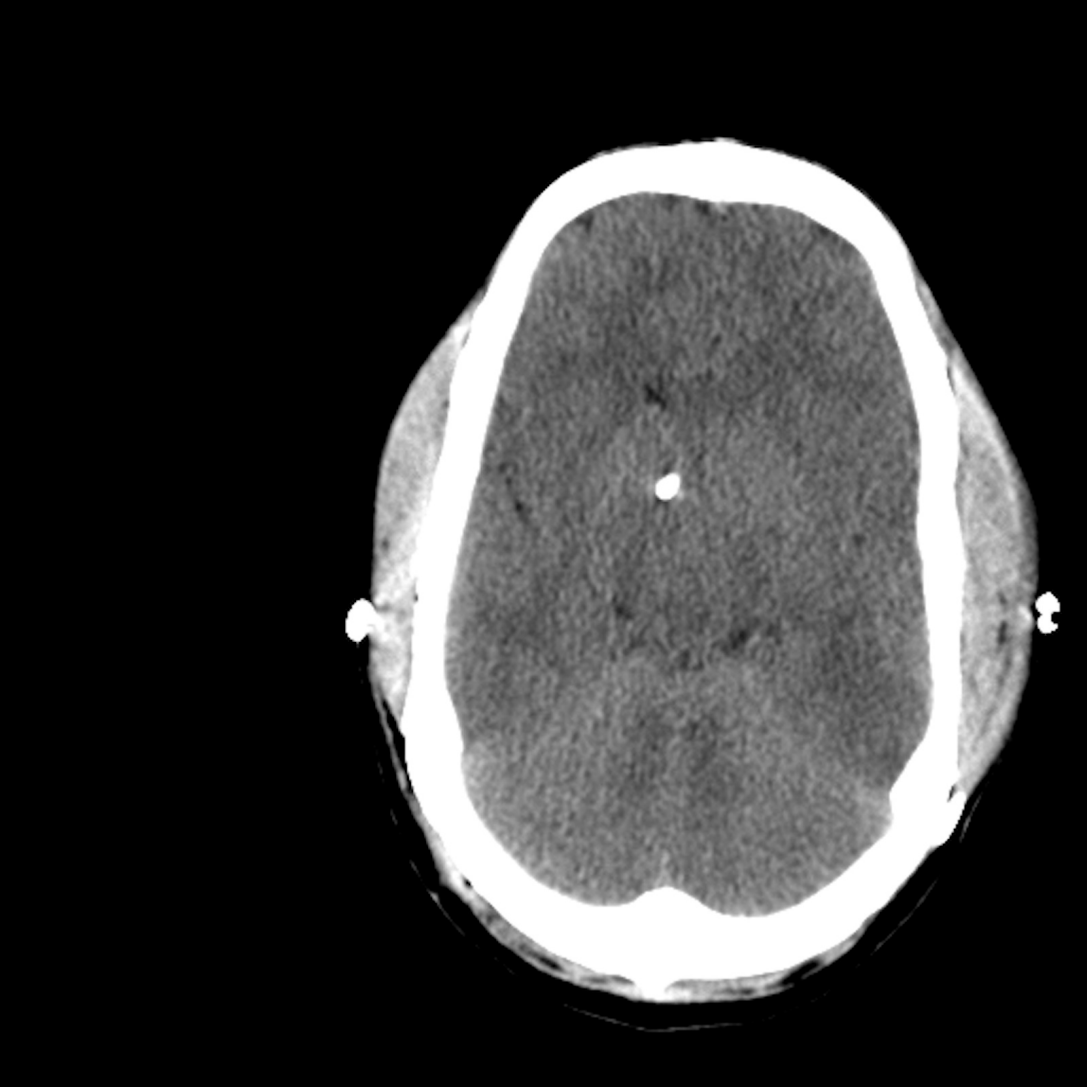
Case 2 pre-expansion

**Figure 4:**
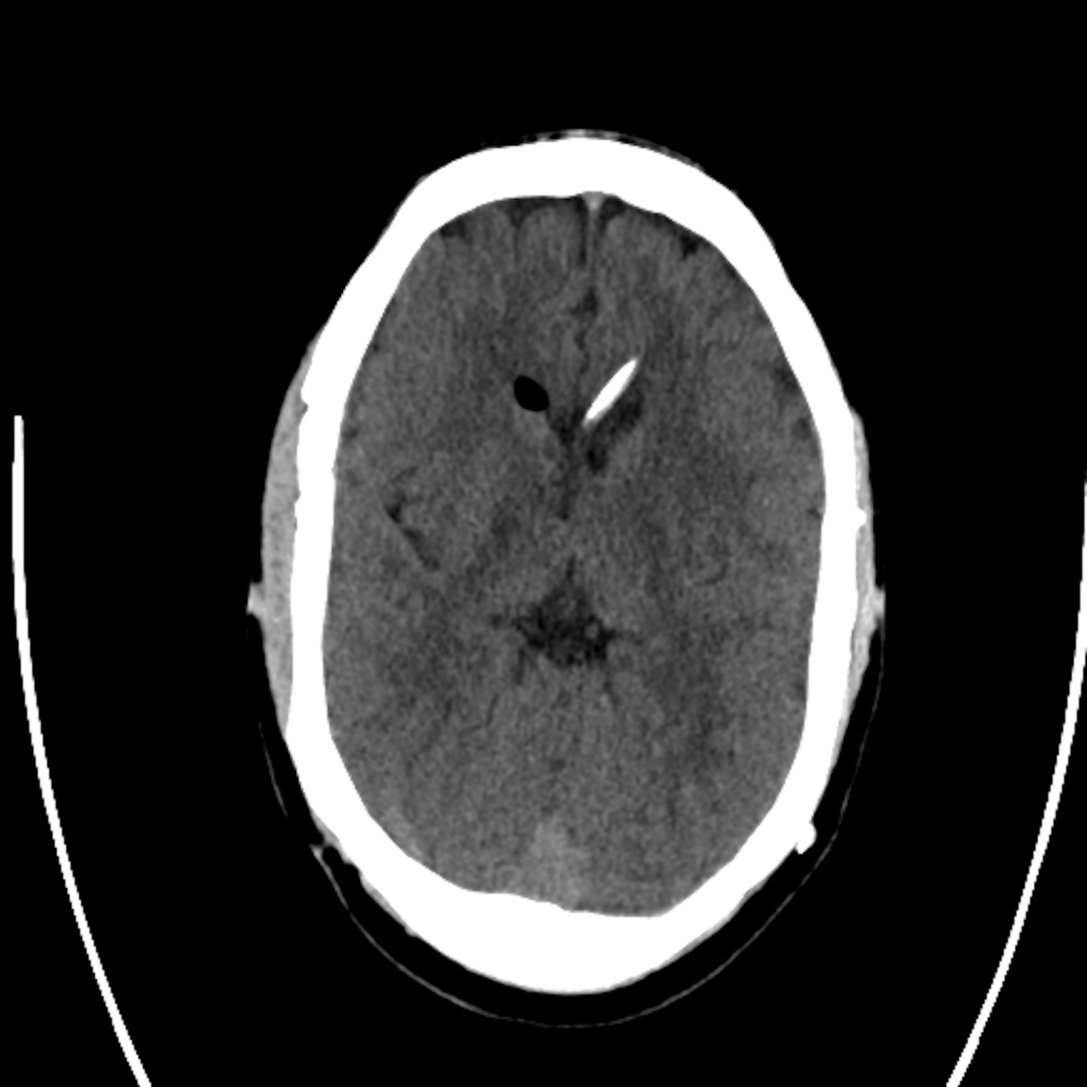
Case 2 immediately post-expansion

He presented to the University of Colorado Hospital at 26 years of age with severe headache. Computed tomography demonstrated closure of all cranial sutures and slit-like ventricles (Figure 3). His head circumference was 38 cm. The patient underwent cranial vault expansion by distraction osteogenesis with each frontoparietal region translated laterally by approximately 1.5 cm (Figure 4). His ventriculoperitoneal shunt was revised approximately one week after distractor implantation for symptoms of headache, nausea, lethargy, and CT that showed enlargement of his right lateral ventricle. The distractors were removed at 70 days due to pain at the site of the left frontal pin and possible infection.

The patient experienced a fall, possibly related to gait instability, three years after vault expansion, causing a subdural hematoma of approximately 5 millimeter (mm) thickness, which resolved without surgery. He has undergone eight surgeries since vault expansion to reposition his ventricular catheters and implant intracranial pressure monitors with little impact on his symptoms. The patient has experienced no episodes of profound headache, vomiting or impaired consciousness since the vault expansion but does complain of occasional headaches of variable intensity, almost always localized over the shunt tract.

Case 3

**Figure 5:**
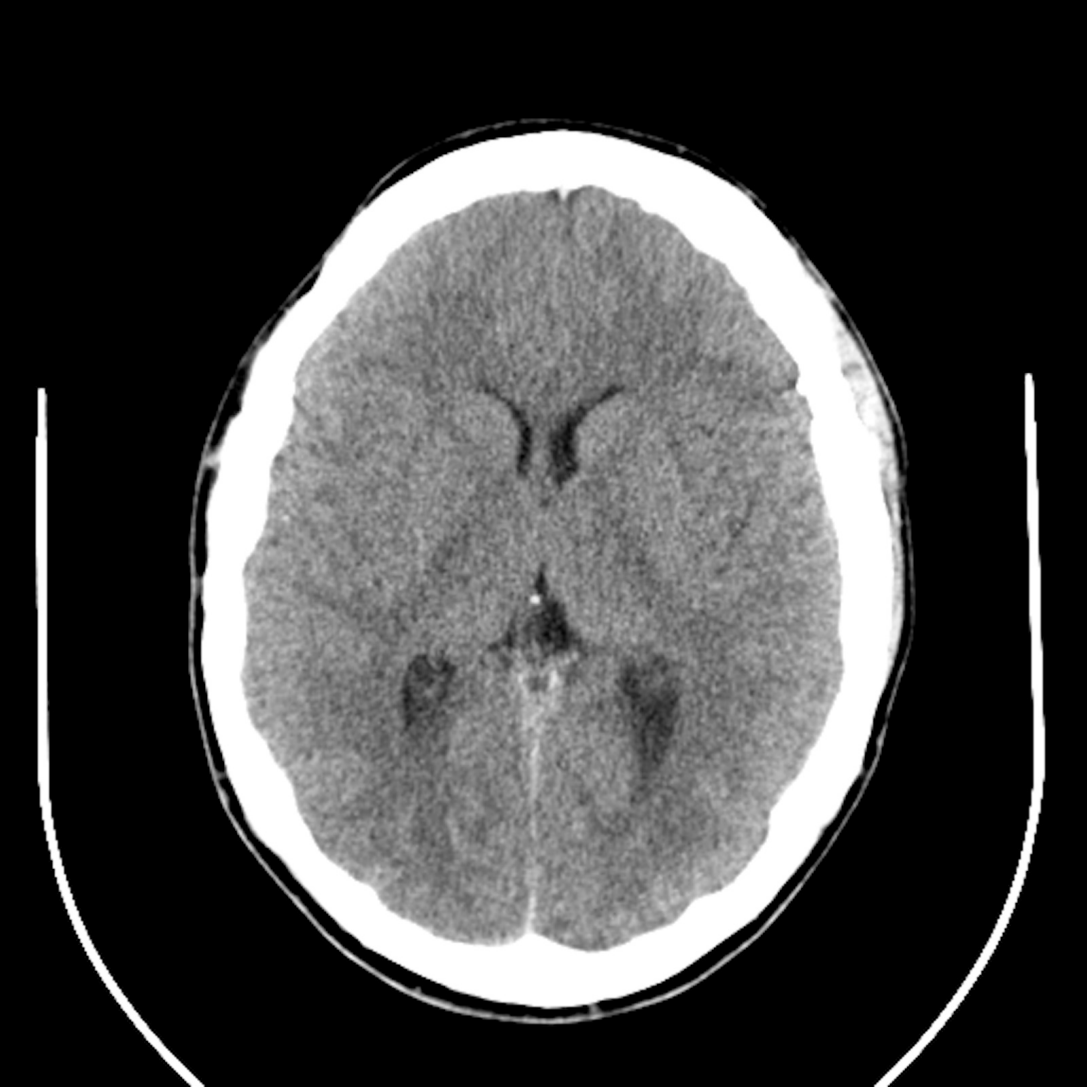
Case 3 pre-expansion

A 21-year-old male presented at the University of Colorado Hospital with two years of intermittent but severely debilitating headaches, nausea, and blurred vision. His past medical history included surgery for sagittal synostosis in infancy. The patient’s past medical history was significant for hypogonadism, adrenal insufficiency, asthma, and factor XII deficiency. Neuroimaging at presentation showed brain parenchyma that appeared tightly compacted within a relatively small cranial vault (head circumference 58.4 cm) and his ventricles were very small (Figure 5). A Camino pressure monitor revealed, during overnight observation, intracranial pressures between 14.0 and 17.6 mmHg while supine.

**Figure 6:**
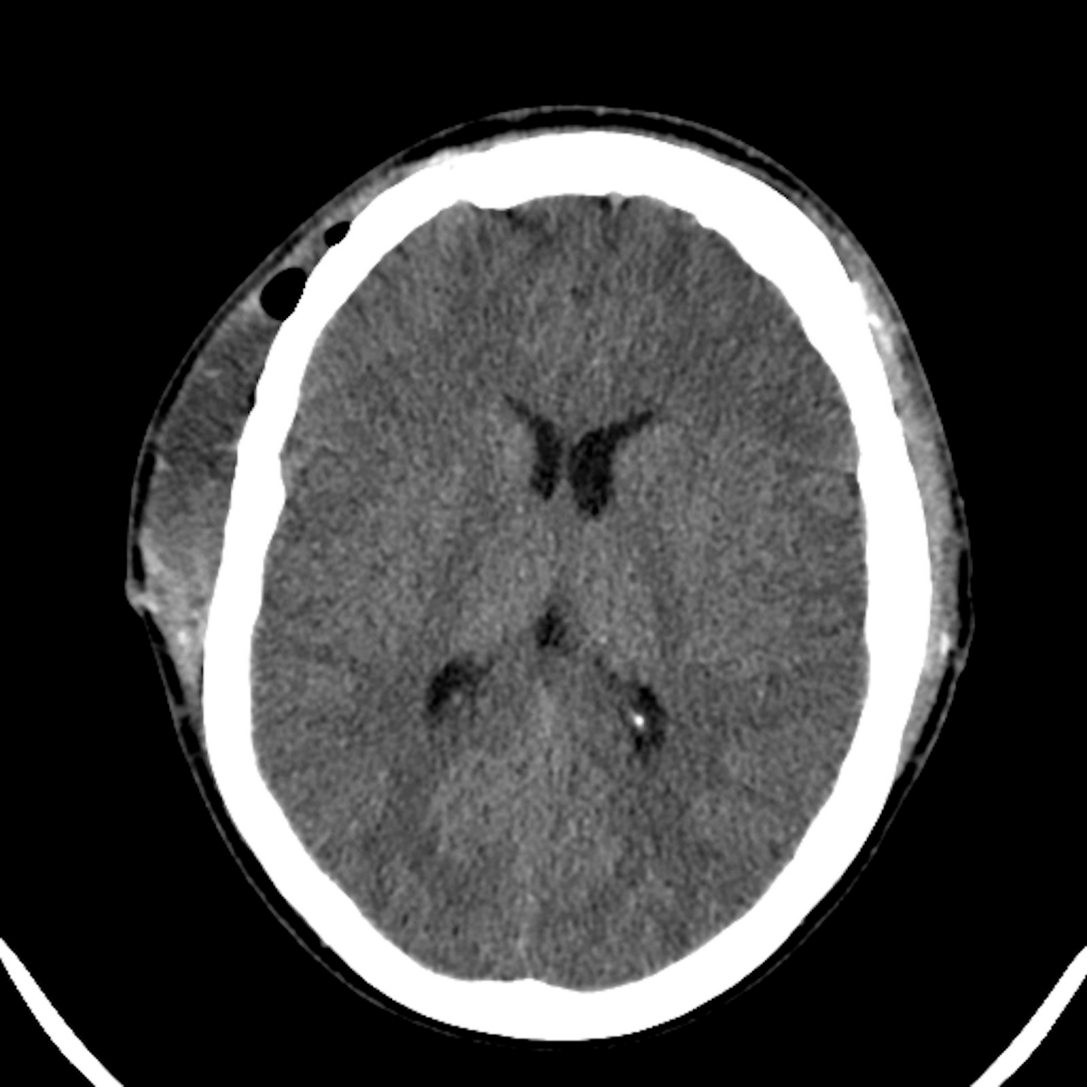
Case 3 immediately post-expansion

The patient underwent bilateral frontoparietal cranial vault expansion of 15 mm over 15 days (1.5 cm bilaterally), followed by 30 days of consolidation (Figure 6). The consolidation period was shorter than planned due to methicillin-resistant Staphylococcus aureus infection at the distractor sites. When the distractors were removed, titanium plates were implanted to stabilize the positions of the cranial bone fragments. Fifty days later all hardware was removed and bony irregularities were remodeled. The patient experienced complete resolution of headaches during the first few days of vault expansion and they never reappeared.

## Discussion

Three adults with severe and worsening headaches and lethargy benefited greatly from cranial vault expansion. Before expansion all three were significantly disabled and were spending much of their life in bed. One was a 20-year old who had undergone surgery for scaphocephaly in infancy and began in adolescence to have severe and progressively worsening headaches and lethargy but no cause had been identified. His evaluation at University of Colorado Hospital revealed multisutural synostosis, small ventricles, and very high intracranial pressure despite a head circumference at the 90th percentile. These findings were interpreted as consistent with the diagnosis of craniocerebral disproportion caused by multisutural synostosis, which occurred as a consequence of surgery in infancy for craniosynostosis [2,3,6,8].

Two patients ages 26 and 30, both with cerebral spinal fluid (CSF) shunts implanted in infancy, had experienced many years of repeated episodes of high intracranial hypertension, which had always been attributed to CSF shunt failures. Each patient had undergone many shunt revisions and the dominant recorded explanations for the shunt failures were obstructions of ventricular catheters by choroid plexus or brain tissue. Their ventricles, however, had been consistently small. Starting in adolescence, the shunt revisions provided only fleeting relief and the revisions became more frequent and achieved progressively shorter periods of benefit. The cause of the repeated shunt failures in adulthood had been attributed to slit-ventricle syndrome. When evaluated at University of Colorado Hospital for chronic severe headaches these patients were again noted to have very small ventricles and multisutural synostosis. Each was thought to have intracranial hypertension in the presence of a normally functioning CSF shunt. This combination of symptoms and neuroimaging was consistent not only with slit-ventricle syndrome but also with craniocerebral disproportion. The intracranial hypertension related to craniocerebral disproportion had almost certainly been present for very many years.

The term slit-ventricle syndrome is used for patients with CSF shunts and a constellation of symptoms and signs that include frequent and severe headaches, lethargy, intracranial hypertension, and small ventricles [2,3]. These patients have a history of having CSF shunts with low outflow resistance from infancy [2-4]. The chronic exposure to abnormally low intracranial pressure during infancy and childhood is thought to predispose to multisutural synostosis, which halts further expansion of their cranial vaults at an age when the brain is still growing [2-5,7,10]. Patients with slit-ventricle syndrome often undergo repeated shunt revisions with frustratingly little success [2,3]. The walls of their lateral ventricles and choroid plexus become tightly pressed against the ventricular catheter, often occluding its holes and causing shunt failure [11]. Shunt revision clears tissue from the holes in the catheter and may include repositioning the catheter within the ventricle but does not expand the ventricles away from the catheter and therefore does not address the cause of the repeated shunt failures. Therefore patients with slit-ventricle syndrome and CSF shunts who have multisutural synostosis have insufficient cranial space for brain and CSF and have insufficient ventricular space to allow long-term CSF shunt patency.

Surgeries performed in infancy for two very different disorders, craniosynostosis and hydrocephalus, resulted in the same secondary patho-anatomical disorder, multisutural craniosynostosis, which locked the size of their intracranial vaults. The multisutural craniosynostosis then led to craniocerebral disproportion as the enclosed brains grew larger. Cranial vault expansion decompressed the brain parenchymas and provided more space for CSF, thereby improving the overall quality of life [4,9]. The fact that these three patients benefited greatly from cranial vault expansion is consistent with the position that all had insufficient cranial space before their cranial vaults were expanded. There have been other reports of the beneficial effect of cranial vault expansion in patients with slit-ventricle syndrome, craniocerebral disproportion, and osteopetrosis [3-6,8,10,12,13]. In retrospect, the patients in this report would have benefited greatly from cranial vault expansion many years earlier.

The clinical features associated with craniocerebral disproportion, craniostenosis, and slit-ventricle syndrome have extensive commonality. The term ‘craniocerebral disproportion’ focuses attention on the mismatch between vault size and brain size, whereas ‘craniostenosis’ refers to the existence of a tightly fitting cranial vault. The term ‘slit-ventricle syndrome’ focuses attention on small ventricles that expand minimally in response to intracranial hypertension in patients with CSF shunts. Craniocerebral disproportion is commonly present in patients with multisutural synostosis and craniostenosis and, in patients with CSF shunts, may be categorized as slit-ventricle syndrome. Many, perhaps all, patients with slit-ventricle syndrome could be more accurately designated as having craniocerebral disproportion [1,2,4,14,15]. It is the opinion of these authors that the term craniocerebral disproportion subsumes the other two terms and communicates a common patho-anatomic disorder, which should not be restricted by age, head circumference or presence or functionality of a CSF shunt.

Most surgeries done to expand the cranial vault in children are done to alleviate intracranial hypertension resulting from craniosynostoses, regardless of cause [4,15]. It is highly likely that most if not all patients with slit-ventricle syndrome have craniocerebral disproportion as an often unrecognized anatomic sine qua non and perhaps a few of these patients do not require a shunt. There is a case report of an infant with osteopetrosis and a CSF shunt that required shunt revisions for slit-ventricle syndrome but, after cranial vault expansion, no longer required a shunt [16]. The critical patho-anatomic abnormality in the patients of this series is, regardless of label applied, insufficient intracranial volume.

Craniocerebral disproportion is rarely considered as a cause for chronic severe headaches in adults but this pathophysiologic state is probably more common than recognized in patients who have either undergone surgery for craniosynostosis or have had CSF shunts implanted in infancy. Both categories are at risk for multisutural synostosis and thus craniocerebral disproportion. Cranial vault expansion, although not simple, risk free, or inexpensive, would likely greatly improve these patients’ quality of life with avoidance of years of suffering and expensive, often minimally successful shunt revisions.

## Conclusions

Craniocerebral disproportion should be considered in adult patients as well as children who have severe headaches, small cerebral ventricles, and multisutural synostosis, regardless of cause. There is a treatment for slit ventricle syndrome other than multiple futile shunt revisions. Cranial vault expansion is a very effective treatment.
